# Development of Epigallocatechin-Loaded Water Caltrop Starch Aerogels: Structure, Functional Properties and In Vitro Release Behavior

**DOI:** 10.3390/foods15183320

**Published:** 2026-09-19

**Authors:** Xueying Dong, Jiachen Wu, Tianle Yao, Pingze Xu, Sijia Zeng, Yiyuan Chang, Jiajun Liu, Hongqiang Wang, Xiaoke Zhang, Li Li, Mengtian Huang, Yu Han

**Affiliations:** 1 Hubei Key Laboratory of Resource Utilization and Quality Control of Characteristic Crops, College of Life Science and Technology, Hubei Engineering University, Xiaogan 432000, China; xueyinghbtu@163.com (X.D.); wujiachenhb@163.com (J.W.); y4201tl@163.com (T.Y.); yiyun2027xu@163.com (P.X.); anonymous11yy@163.com (S.Z.); meikele2026@163.com (Y.C.); redvelvet20142024@163.com (J.L.); whq11354qiang@163.com (H.W.); mianmiantu522@163.com (X.Z.); lili2023new@163.com (L.L.); 2 National “111” Center for Cellular Regulation and Molecular Pharmaceutics, Hubei University of Technology, Wuhan 430068, China; 3 College of Food Science & Technology, Huazhong Agricultural University, Wuhan 430070, China

**Keywords:** water caltrop starch, aerogel, epigallocatechin, controlled release, biodegradable packaging, polyphenol stabilization

## Abstract

The increasing demand for sustainable food packaging has generated interest in biodegradable carriers for bioactive chemicals. This study created porous aerogels from water caltrop starch (WCS) to encapsulate and regulate the release of epigallocatechin (EGC), a sensitive natural polyphenol. WCS aerogels with different EGC concentrations (0.6–2.5 g per 2.5 g starch) were produced using sol–gel processing and freeze-drying. Structural investigations (FTIR, XRD) suggested bonding interactions between WCS and EGC, which stabilized the polyphenol and affected crystallinity. Scanning electron microscopy demonstrated that an appropriate EGC loading (1.8 g) facilitated a well-connected porous network, improving water and oil retention. Rheological and textural investigations demonstrated that the addition of EGC enhanced the gel network, leading to increased mechanical properties. In vitro release tests revealed a sustained, diffusion-controlled release profile over 10 days, with higher EGC loading resulting in enhanced retention. The aerogels demonstrated concentration-dependent antibacterial efficacy against *E. coli* and *S. aureus*, and exhibited preliminary degradability in soil and water. These findings suggest that WCS-based aerogels represent a potential biodegradable delivery system for polyphenols, demanding additional research for functional food packaging.

## 1. Introduction

The extensive use of petroleum-based plastics in food packaging has generated serious environmental concerns due to their poor biodegradability and long-term accumulation in ecosystems [1,2]. Simultaneously, consumer demand for minimally processed foods with extended shelf life has stimulated the development of active packaging systems capable of delivering functional compounds to enhance food quality and safety [3,4]. Consequently, biodegradable packaging materials derived from renewable biopolymers have attracted increasing attention as sustainable alternatives to conventional plastics.

Aerogels are a distinctive category of solid substances characterized by a three-dimensional, extensively porous framework, generally created by substituting the liquid solvent in a gel with air or gas while maintaining the original gel structure. This is typically accomplished using specialized drying methods, like supercritical drying or freeze-drying, which inhibit capillary forces from compromising the porous structure and allow the liquid phase to be replaced by gas [5]. Due to their unique structure, aerogels possess an array of extraordinary characteristics, including ultrahigh porosity, minimal density, extensive specific surface area, and superior thermal insulation properties [6,7]. Aerogel technology was first created using inorganic substances like silica, but it has gradually evolved to incorporate biopolymer-based systems, motivated by an increasing demand for sustainable and biodegradable functional materials in food and biomedical sectors.

In the realm of food packaging, aerogels have numerous practical benefits compared to traditional plastic materials. In contrast to conventional plastic packaging, which mostly functions as a passive barrier, aerogel-based packaging can proactively aid in food preservation through various mechanisms [8]. Firstly, aerogels’ extensively porous architecture with substantial specific surface areas facilitates the entrapment and regulated discharge of antibacterial and antioxidant substances, offering proactive defense against microbial degradation and oxidative damage [8,9]. Secondly, aerogels can serve as superabsorbent materials to control exudates from fresh fruits and meats, thus diminishing moisture buildup that fosters bacterial proliferation [9]. Thirdly, the exceptionally lightweight and thermally insulating properties of aerogels render them ideal for use as cushioning pads for mechanical safeguarding and as insulation materials for temperature-sensitive chilled or frozen products [9]. Moreover, aerogels can be engineered as smart labels or indicators that react to pH fluctuations or volatile amines for immediate freshness assessment [9]. Significantly, aerogels composed of biopolymers are biodegradable and sourced from renewable materials, mitigating the environmental impact associated with traditional petroleum-derived plastics [8,9]. Starch-derived aerogels have proven to be effective substitutes for conventional plastic packaging due to their superior biodegradability, biocompatibility, and adjustable mechanical characteristics [10,11,12]. The integration of dynamic functionality, minimal protection, and total biodegradability establishes aerogels as a revolutionary medium for future sustainable food packaging [8].

Water caltrop (*Trapa bispinosa* Roxb.) is an aquatic crop widely cultivated in Asia, particularly in China. In contrast to traditional starch sources typically utilized in aerogels, such as corn, rice, potato, and cassava starches [13], WCS shows unique gelatinization and retrogradation characteristics, along with a moderate-to-high amylose concentration, which might offer particular benefits for gel network formation and freeze-drying performance [14]. Previous investigations have primarily focused on the extraction, physicochemical properties, and food-processing applications of water caltrop starch (WCS) [15,16]. Nevertheless, its potential as a structural matrix for aerogel fabrication and as a carrier for bioactive polyphenols remains largely unexplored. The development of WCS-based aerogels would not only expand the utilization of this underexploited starch resource but also provide new opportunities for high-value applications in sustainable food packaging [5].

Epigallocatechin (EGC), a major catechin naturally occurring in tea leaves, has attracted considerable attention because of its potent antioxidant, antimicrobial, and health-promoting activities [17]. Owing to these properties, EGC is considered a promising active ingredient for food preservation systems. However, its practical application is limited by poor environmental stability. EGC is highly susceptible to oxidation, degradation, and loss of bioactivity when exposed to oxygen, light, moisture, and elevated temperatures [18]. Encapsulation within biopolymeric matrices has therefore been proposed as an effective strategy to protect EGC from environmental stress and achieve sustained release during storage and application.

Recent studies have demonstrated that polyphenols can interact with starch molecules through hydrogen bonding, hydrophobic interactions, and molecular entanglement, resulting in significant modifications of starch structure and functionality. These interactions may influence gelation behavior, crystallinity, pore morphology, mechanical properties, and release kinetics of starch-based delivery systems [19,20,21]. Although starch–polyphenol complexes have been extensively investigated in films, hydrogels, and nanoparticles [22], the relationship between molecular interactions, aerogel microstructure, functional properties, and controlled release behavior in WCS-based aerogel systems remains poorly understood. In particular, the influence of EGC incorporation on the formation and performance of WCS aerogels has not yet been systematically investigated.

Therefore, the objectives of this study were to develop EGC-loaded WCS aerogels through a freeze-drying process and to elucidate the effects of EGC incorporation on their structural characteristics, physicochemical properties, mechanical performance, and release behavior. Fourier-transform infrared spectroscopy (FTIR), X-ray diffraction (XRD), scanning electron microscopy (SEM), rheological analysis, and texture profile analysis were employed to investigate the structure–property relationships of the aerogels. The developed EGC-loaded WCS aerogels can be fabricated into various active packaging formats, such as cushioning pads, absorbent inserts, interleaving sheets, or stand-alone sachets for fresh meat, seafood, and perishable produce. The incorporation of EGC provides continuous antioxidant and antimicrobial protection through controlled release, while the porous structure enables absorption of liquid exudates to suppress microbial growth. Unlike conventional plastic packaging, these aerogels combine active preservation functions with superabsorbency, cushioning, thermal insulation, and complete biodegradability, positioning them as multifunctional sustainable alternatives for active food packaging [5]. Furthermore, the controlled release behavior, antibacterial activity, and environmental degradability of the developed materials were evaluated to assess their potential application as sustainable active packaging systems. This work provides new insights into the design of starch-based aerogel carriers for the stabilization and delivery of natural bioactive compounds while promoting the high-value utilization of water caltrop starch resources.

## 2. Methods and Materials

### 2.1. Materials and Instruments

Fresh water caltrop was harvested from Hanchuan, Hubei, China. Epigallocatechin (EGC, ≥98%, HPLC grade) was procured from Shanghai Yuanye Bio-Technology Co., Ltd. (Shanghai, China). Seventy percent ethanol, Folin–Ciocalteu reagent, 7.5% sodium carbonate solution, and phenolphthalein were acquired from Sinopharm Chemical Reagent Co., Ltd. (Shanghai, China). Ultrapure water (resistivity ≥ 18.2 MΩ·cm) was utilized in all studies. Sodium hydroxide was acquired from Sinopharm Chemical Reagent Co., Ltd. All compounds were of analytical grade unless specified otherwise. The soil utilized for the exposure investigation was collected from the surface layer (0–20 cm depth) of an experimental field in Xiaogan (Hubei, China) and was sieved through a 2 mm mesh to remove plant remnants and stones before application.

The apparatus employed included a vacuum freeze dryer (FD-1, Boyikang, Beijing, China), scanning electron microscope (SEM, SU8010, Hitachi, Tokyo, Japan), Fourier-transform infrared spectrometer (FTIR, Nicolet iS50, Thermo Scientific, Waltham, MA, USA), X-ray diffractometer (XRD, D8 Advance, Bruker, Bremen, Germany), rotational rheometer (MCR 302, Anton Paar, Graz, Austria), texture analyzer (TA.XT plus, Stable Micro Systems, Godalming, UK), and DS-200 Colorimeter (Caipu Technology, Hangzhou, China).

### 2.2. Extraction of WCS

WCS was obtained by a modified alkaline-ultrasonication technique. Homogenized peeled Water Caltrop tubers were suspended in a 0.4% NaOH solution at a solid-to-liquid ratio of 1:20 (g/mL). The suspension was sonicated (400 W, 35 °C) for 20 min and then filtered through a 100-mesh screen. The filtrate was centrifuged at 4000× *g* for 15 min, and the precipitate was washed with deionized water until a neutral pH was achieved. WCS powder was desiccated at 40 °C and stored in a desiccator [23]. The iodine colorimetric method was applied to determine the amylose concentration. The absorbance was measured at 635 nm using a UV spectrophotometer (Metash V-5600, Nanjing, China).

The extraction rate of WCS was calculated according to the following formula:

(1) $$\mathrm{Extraction}\;\mathrm{rate}\;(\%)\;=\frac{W_{1}}{W_{0}}\times 100\%$$

where W_0_ is the dry weight of the water caltrop powder used and W_1_ is the dry weight of the final WCS powder obtained after drying at 40 °C to constant weight.

### 2.3. Structural Analysis of WCS

#### 2.3.1. Fourier Transform Infrared (FTIR) Spectroscopy

Approximately 1 mg of WCS powder was combined with 100 mg of anhydrous KBr and meticulously milled. The mixture was compacted for 2 min in a transparent pellet at a pressure of 1.5 MPa. The FTIR spectra were obtained from 4000 to 500 cm^−1^ with a resolution of 2 cm^−1^ and 64 scans, using air as the background. Characteristic peaks were examined to assess the molecular functional groups [4].

#### 2.3.2. X-Ray Diffraction (XRD)

WCS powder was evenly distributed on the sample stage. XRD patterns were obtained using Cu-Kα radiation (λ = 1.5406 Å) at 40 kV and 40 mA. The scans were conducted at 10–80° (2θ) at a rate of 2°/min with a step increment of 0.02°. Crystallinity was determined via the Segal method based on peak intensities.

#### 2.3.3. Scanning Electron Microscopy (SEM)

WCS powder was affixed to conductive adhesive tapes and subsequently coated with gold. SEM pictures were acquired at an accelerating voltage of 5 kV with magnifications of 1000×, 2000×, and 3000× to examine particle morphology and dimensions [4].

### 2.4. Rheological Properties of WCS

Dynamic rheological measurements were conducted utilizing a rotational rheometer featuring a 40 mm diameter, 1° cone-plate geometry (gap: 0.1 mm) at 25 °C. Frequency sweep experiments (0.01–20 rad/s) were performed inside the linear viscoelastic region (strain = 1%) to ascertain the storage (G′) and loss (G″) moduli.

The instrument was preheated and permitted to stabilize at 25 °C. The rheological characteristics of the cooled WCS paste were examined utilizing a rotational rheometer with a 40 mm diameter, 1° cone-plate configuration. An aliquot sample was evenly distributed on the lower plate, and the upper geometry was adjusted to a gap of 0.1 mm. The surplus sample at the edge was meticulously removed using a lint-free tissue to avert edge drying and maintain a uniform testing environment. Dynamic oscillatory frequency sweeps were conducted within the established linear viscoelastic zone (strain = 1%) across an angular frequency range of 0.01 to 20 rad/s. The storage modulus (G′) and loss modulus (G″) were measured as functions of frequency [23].

### 2.5. Preparation of Aerogels

#### 2.5.1. Preparation of WCS Aerogels

To enhance the starch concentration for aerogel formulation, three formulations using different WCS quantities (2.0, 2.5, and 3.0 g in 50 mL of deionized water) were developed and analyzed. A homogeneous suspension of starch (2.0 g, 2.5 g, and 3.0 g WCS in 50 mL deionized water, respectively) was boiled for 10 min at 95 °C with constant stirring until fully gelatinized. Following the method proposed by Tawiah et al. (2023) and making minor adjustments, the paste was placed into 12-well polystyrene molds and pre-frozen at −20 °C for 12 h to facilitate the development of ice crystals [7]. The pre-frozen specimens were promptly moved to the freeze-dryer and subjected to freeze-drying under vacuum conditions (cold trap temperature: −55 °C, <10 Pa) for 24 h. Dried aerogels were stored in a desiccator at room temperature before examination.

#### 2.5.2. Preparation of EGC-Loaded Aerogels

The optimal WCS concentration was used for all EGC-loaded aerogel formulations. EGC was added at four concentrations (0.6, 1.2, 1.8, and 2.5 g) to the WCS paste immediately after gelatinization. The liquid was homogenized for 2 min to achieve uniform dispersion and then cast into molds at 2 mL per well. The samples were cryopreserved at −20 °C for 12 h to facilitate ice crystal templating, followed by freeze-drying under the conditions specified in Section 2.5.1.

### 2.6. Characterization of EGC-Loaded Aerogels

#### 2.6.1. Physicochemical Evaluation

FTIR and XRD studies of aerogel powders were as outlined in Section 2.3 to assess molecular interactions and alterations in crystallinity subsequent to EGC loading.

#### 2.6.2. Morphological

The surface morphology was examined via SEM following the protocol outlined in Section 2.3.3. SEM images were magnified at 40×, 150×, and 200× to examine aerogel morphology. To evaluate the influence of EGC incorporation on pore structure, the microstructural characteristics of the porous network were examined with ImageJ software 2025 to calculate porosity.

#### 2.6.3. Texture Profile Analysis

Before conducting texture profile analysis, all cylindrical aerogel samples were equilibrated at 25 °C and 55% relative humidity (RH) for 24 h. All assessments were subsequently conducted under identical environmental parameters (25 °C, 55% RH). Cylindrical aerogel specimens were subjected to two consecutive compression cycles (two-cycle TPA) using a texture analyzer equipped with a P/50 flat probe at a rate of 1 mm/s, 50% strain, and a 5-s interval between successive compression cycles. Hardness and springiness were assessed using standard Texture Profile Analysis (TPA) techniques [23].

#### 2.6.4. Water and Oil Retention Capacities (WHC/OHC)

The ground and sieved aerogel powder (0.5 g, passed through a 40-mesh sieve) was combined with 10 mL of deionized water or soybean oil, vortexed for 30 s, and permitted to equilibrate at 25 °C for 30 min. Samples underwent centrifugation at 5000× *g* for 15 min, after which the supernatant was discarded [4]. WHC and OHC were computed as follows:

(2) $$\mathrm{WHC}/\mathrm{OHC}(g/g)=\frac{W_{\mathrm{hydrated}}-W_{\mathrm{dry}}}{W_{\mathrm{dry}}}$$

where W*_hydrated_* and W*_dry_* denote the weights of hydrated and dry aerogel, respectively.

#### 2.6.5. Colorimetric Evaluation

The hue of aerogels with differing EGC loadings was assessed utilizing a portable spectrophotometer (D65 illuminant, 10° observer, Cai Pu Technology Co., Ltd., Taizhou, China). L*, a*, and b* measurements were documented at three arbitrary locations per sample [4].

### 2.7. In Vitro Release and Retention of EGC from WCS Aerogels

The in vitro release of EGC from the aerogel matrix was observed over 10 days. Powdered aerogel samples (0.1 g) were suspended in 5 mL of 70% ethanol, vortexed for 30 s, then subjected to sonication for 10 min, ensuring thorough extraction of released EGC. After centrifugation at 5000× *g* for 15 min, the supernatant was collected for analysis. The concentration of EGC in the supernatant was evaluated using the Folin–Ciocalteu reagent. A total of 0.5 mL of the supernatant was combined with 2.5 mL of Folin–Ciocalteu reagent (diluted 1:10 with deionized water) and permitted to sit for 5 min. Following this, 2.0 mL of a 7.5% (*w*/*v*) sodium carbonate solution was added, and the combination was allowed to incubate in darkness at 25 °C for 60 min. The absorbance was measured at 765 nm utilizing a UV-Vis spectrophotometer (Metash V-5600, Nanjing, China), with a blank composed of 70% ethanol developed corresponding to the identical protocol [24]. A calibration curve was constructed using gallic acid as the standard (0–50 μg/mL), yielding the linear regression equation. EGC retention rate (Rt) and EGC loading rate (Rc) were computed in accordance with Equations (3) and (4), respectively.

(3) $$R_{t}(\%)=\frac{C_{t}}{C_{1}}\times 100\%$$

(4) $$R_{C}=\frac{C_{0}}{C}\times 100\%$$

where C_t_ is the EGC concentration in the release medium at a given time t (t = 1, 3, 5, 7, or 10 days), C_1_ is the EGC concentration on day 1, C_0_ he EGC concentration measured for the newly formed aerogels, and C is the EGC added concentration. All measurements were performed in triplicate, and results were expressed as mean ± standard deviation.

### 2.8. Antibacterial Efficacy

The antibacterial activity against *Escherichia coli* (*E. coli* ATCC 25922) and *Staphylococcus aureus* (*S. aureus* ATCC 25923) was evaluated using the agar disc diffusion method. Aerogel squares containing 0.6 g, 1.2 g, 1.8 g and 2.4 g EGC and control discs underwent UV sterilization. A bacterial suspension with an optical density of 0.8 at 600 nm was inoculated into LB agar plates. Samples and controls, including negative (sterile water), positive (100 µg/mL ampicillin), and blank aerogel, were positioned on the inoculated agar. Following a 24-h incubation at 37 °C, the diameter of the inhibitory zone was assessed. Experiments were conducted in triplicate.

### 2.9. Evaluation of Exposure

The environmental stability of aerogels prepared with different EGC loading levels (0.6, 1.2, 1.8, and 2.5 g per 2.5 g WCS) was assessed under the specified test conditions. Triplicate samples were entombed in organic soil under natural outdoor settings. Triplicate samples were positioned on an organic soil surface consisting of 80% peat moss, 15% coconut coir, and 5% perlite, exhibiting an organic matter content ≥ 60%, a total NPK concentration of 2.5%, and a pH range of 5.5 to 6.5. Samples were collected at predetermined intervals (10, 20, 30, 40 days), photographed to record morphological alterations, desiccated, and weighed to assess mass loss. The exposure test was conducted in regulated laboratory conditions without additional water introduction. Before weighing, the samples were gently purified using lint-free paper to remove surface-bound soil particles. A water solubility test was performed by submerging samples in deionized water at 25 °C and observing their physical disintegration over 24 h to simulate the degradation behavior in the presence of water under natural environmental conditions.

### 2.10. Statistical Analysis

All experiments were performed in three independent replicates (*n* = 3) using separately prepared batches, and the data are expressed as mean ± standard deviation (SD). One-way analysis of variance (ANOVA) was utilized to identify significant differences across the groups, subsequently accompanied by SPSS 2026 testing for pairwise comparisons. Statistical analyses were conducted utilizing OriginLab OriginPro 2022 software (OriginLab Corporation, Northampton, MA, USA). Mean values were computed for all measurements, and significant differences across treatments were assessed at a significance level of *p* < 0.05.

## 3. Results

### 3.1. Extraction of WCS

Following single-factor experiments to determine the appropriate ranges of NaOH concentration, material-to-liquid ratio, extraction temperature, extraction duration, and ultrasonic power, response surface methodology (RSM) was utilized to enhance the extraction rate of WCS (Figure S1). According to the quadratic regression model, the ideal conditions were determined as follows: NaOH concentration, 0.4%; material-to-liquid ratio, 1:20 g·mL^−1^; ultrasonic temperature, 35 °C. Given these parameters, the model forecasted a peak extraction rate of 69.315% (Figure S1). To authenticate the model, three separate replicate verification experiments were conducted under ideal conditions, and the extraction rate was determined using the formula provided in the Section 2. The verified average extraction rate achieved from the tests was 65.16 ± 0.85%. The response surface depicted in Figure S1 demonstrated a significant rise followed by a flat region around the optimal zone, indicating a somewhat extensive operational range. The experimental result (65.16%) was slightly lower than the expected value (69.315%); nevertheless, the relative error was approximately 6.0%, which is considered acceptable (<10%) for RSM-based optimization. This confirms that the regression model accurately depicts the authentic extraction process and that the optimized settings are reliable and stable.

The amylose concentration considerably influences the physicochemical characteristics of starch. According to the standard curve for amylose (y = 0.3467x − 0.046, R^2^ = 0.9988, Figure S2), the amylose content in WCS was determined to be 20.31 ± 0.20%.

### 3.2. Rheological Properties of WCS Gels

To investigate the effect of starch concentration on the viscoelastic properties of WCS gels, three formulations were prepared by dispersing 2.0 g, 2.5 g, and 3.0 g of WCS in 50 mL of deionized water, respectively, following the same gelatinization procedure described in Section 2.4.

**Figure 1:**
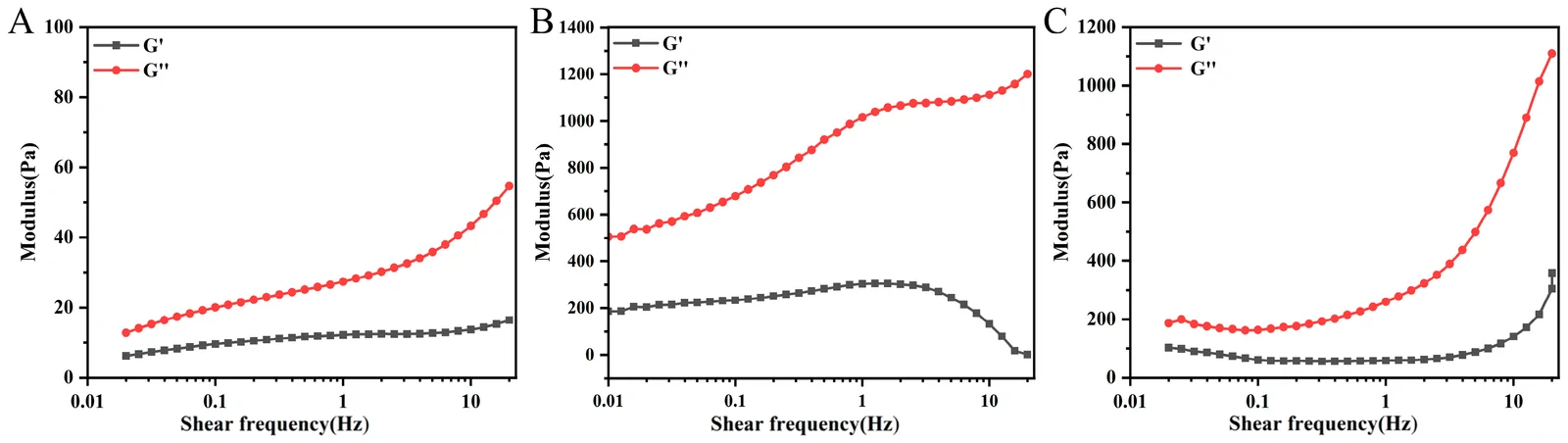
Rheological curves of WCS gels ((**A**): 2.0 g WCS; (**B**): 2.5 g WCS; (**C**): 3.0 g WCS).

The rheological behavior of WCS gels plays a critical role in determining the structural stability of the resulting aerogels. Dynamic frequency sweep measurements were therefore performed to evaluate the viscoelastic properties of starch suspensions with different concentrations (Figure 1). For the 2.0 g WCS formulation (2.0 g/50 mL), the storage modulus (G′) remained higher than the loss modulus (G″) throughout the investigated frequency range, indicating the formation of a weak gel network. However, both moduli exhibited relatively low values and pronounced frequency dependence, suggesting a loosely connected structure with limited resistance to deformation (Figure 1A). Increasing the starch concentration to 2.5 g/50 mL substantially enhanced the viscoelastic properties of the system. The higher G′ and G″ values indicated the formation of a more interconnected and mechanically stable gel network. At low frequencies, the gel exhibited typical elastic-dominant behavior (G′ > G″), whereas a crossover between G′ and G″ occurred at high frequencies, reflecting partial disruption of the network under intense shear conditions. This behavior suggests the formation of a sufficiently strong yet processable gel structure suitable for aerogel fabrication (Figure 1B). In contrast, the 3.0 g/50 mL formulation displayed predominantly viscous characteristics, with G″ exceeding G′ over most of the frequency range. Although the modulus values increased markedly, the system failed to establish a stable elastic network. Excessive starch concentration likely restricted molecular mobility and hindered the development of a homogeneous three-dimensional gel structure, resulting in poor structural stability (Figure 1C). Taken together, the rheological results indicate that the 2.5 g/50 mL formulation provided the optimal balance between network strength and structural integrity. Therefore, this formulation was selected for subsequent EGC loading and aerogel preparation.

### 3.3. Structural Characteristics of WCS Granules and WCS Aerogels

#### 3.3.1. FTIR Analysis

**Figure 2:**
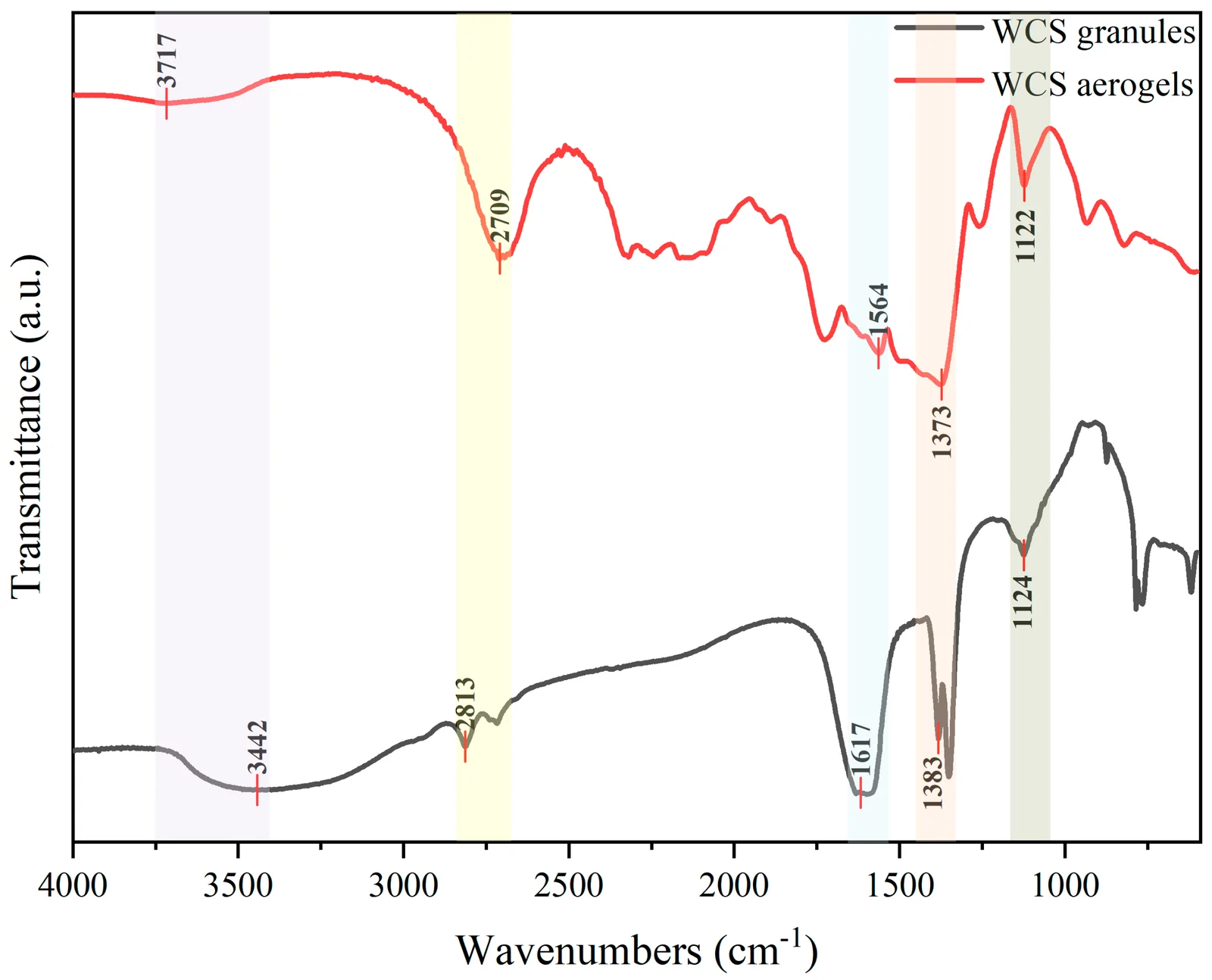
FTIR spectrum of WCS granules and WCS aerogels.

The FTIR spectra of native WCS granules and the corresponding aerogels are presented in Figure 2. The characteristic absorption bands confirmed the successful extraction of WCS and the preservation of its original molecular structure. A broad absorption peak centered at 3442 cm^−1^ was assigned to O–H stretching vibrations associated with intermolecular and intramolecular hydrogen bonding within starch molecules. The band observed at 2813 cm^−1^ corresponded to C–H stretching vibrations of glucose units. The absorption peak at 1617 cm^−1^ was attributed to the bending vibration of adsorbed water, indicating the presence of bound moisture commonly observed in starch granules. In addition, the peaks located at 1383 cm^−1^ were associated with C–H bending vibrations, while the strong absorptions at 1124 cm^−1^ were characteristic of C–O–C glycosidic linkages. These spectral features are consistent with those reported for native starches and collectively confirm the polysaccharide nature of WCS. No additional absorption bands associated with protein or lipid impurities were detected, suggesting that the extraction procedure effectively isolated starch from water caltrop. Notably, the FTIR spectrum of the WCS aerogels retained all the characteristic absorption peaks of WCS granules (e.g., C–H stretching at 2709 cm^−1^, and C–O–C vibrations at 1122 cm^−1^, with no significant shifts observed in the 1370–1390 cm^−1^ region), confirming that the gelatinization and freeze-drying processes did not alter the fundamental chemical structure of the starch matrix.

#### 3.3.2. X-Ray Diffraction Analysis

**Figure 3:**
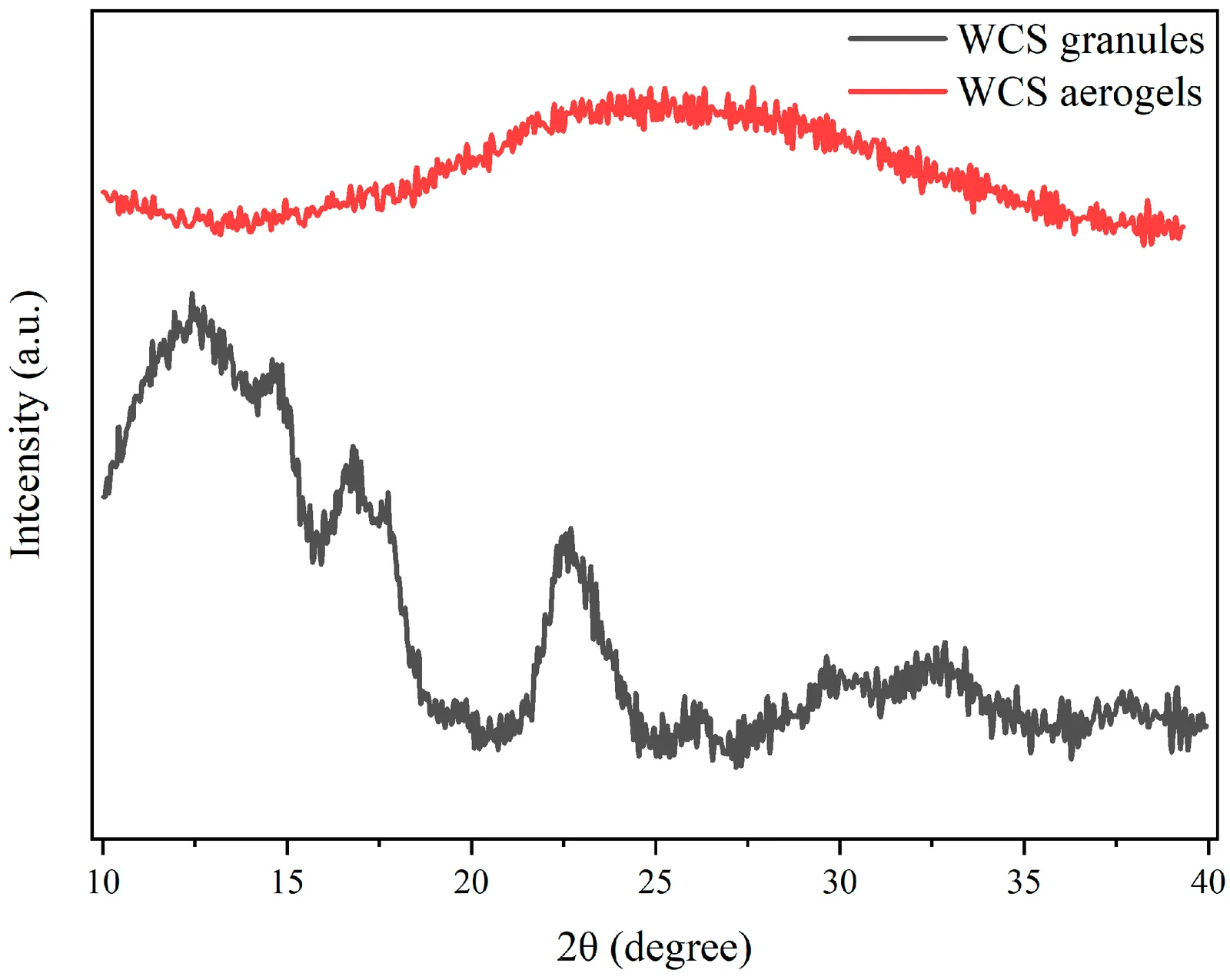
XRD pattern of WCS granules and WCS aerogels.

The crystalline structures of WCS granules and WCS aerogels were investigated by X-ray diffraction (Figure 3). Native WCS granules exhibited distinct diffraction peaks at 2θ values of 15.88°, 16.94°, 17.72°, and 22.76°, which are typical features of A-type starch crystallinity [25]. The grain size of the WCS granules in Jade 9 software was calculated to be 19.74%. Conversely, WCS aerogels exhibited a markedly different diffraction pattern characterized by feeble, wide signals, suggesting that the extensive crystalline organization of native starch was mainly destroyed during gelatinization. This corresponds with the determined crystallinity of the WCS aerogels, which was effectively 0%, demonstrating that the gelatinization process entirely dismantled the organized crystalline structure of the original starch granules. The freeze-drying process did not promote the recrystallization of amylopectin, leading to a typically amorphous structure, confirming that WCS aerogel fabrication process transformed the highly crystalline WCS granules into a largely disordered network structure.

#### 3.3.3. Morphological Characteristics

**Figure 4:**
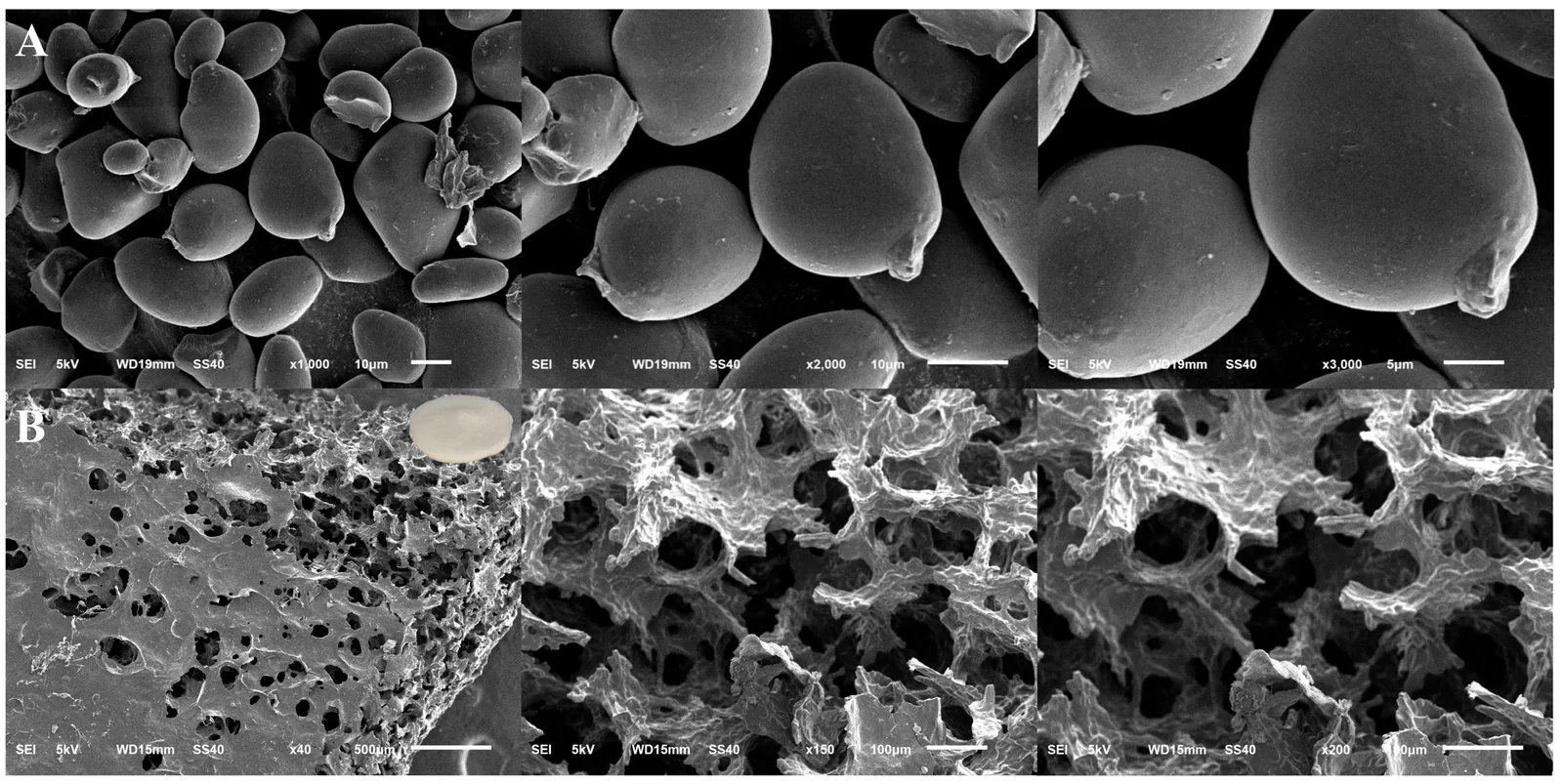
SEM image of WCS granules ((**A**): ×1000, ×2000, ×3000) and WCS aerogels ((**B**): ×40, ×150, ×200).

The morphologies of WCS granules and WCS aerogels were examined by SEM, and the micrographs are presented in Figure 4. WCS granules exhibited predominantly oval and ellipsoidal shapes with smooth and intact surfaces. No obvious fissures, erosion, or structural damage were observed, indicating that the alkaline ultrasonication extraction process caused minimal disruption to the native granule architecture (Figure 4A). The preservation of granule integrity suggested that the extraction conditions were sufficiently mild to remove non-starch components while maintaining the structural characteristics of starch. The observed morphology was comparable to that reported for other native starches and provides further evidence for the successful isolation of high-quality WCS suitable for aerogel preparation.

After gelatinization and freeze-drying, the WCS aerogels had a significantly altered morphology, distinguished by a highly porous three-dimensional structure featuring interconnected pore channels and relatively uniform pore walls (Figure 4B). Throughout the freezing procedure, ice crystals formed and acted as sacrificial molds, subsequently leading to sublimation in a vacuum that generated a unified porosity structure while preserving the gel architecture typical of freeze-dried starch aerogels [26]. The creation of a porous framework represents a complete morphological transformation from the compact, granular arrangement of native starch to a lightweight, open-cell foam architecture. A highly porous framework is advantageous for encapsulating bioactive compounds since it provides several loading sites and improves mass transfer during release processes.

### 3.4. Functional Properties of WCS Aerogels

The functional performance of WCS aerogels was evaluated through their water/oil-holding capacities and mechanical characteristics, both of which are closely related to the integrity of the porous network formed during gelation and freeze-drying. WSC aerogel exhibited high oil retention and low water retention, hence facilitating the subsequent incorporation of fat-soluble polyphenols. As shown in Table 1, the 2.5 g WCS aerogels exhibited a water-holding capacity (WHC) of 5.29 ± 1.65% and an oil-holding capacity (OHC) of 4.51 ± 1.42%. The slightly higher WHC can be attributed to the abundance of hydroxyl groups present in starch molecules, which facilitate hydrogen-bond interactions with water molecules. In contrast, the lower OHC is likely associated with the inherently hydrophilic nature of the starch matrix, which limits interactions with nonpolar oil molecules. Moreover, the relatively compact pore walls and the higher viscosity of soybean oil may restrict oil penetration into the porous network, resulting in reduced oil retention. The moderate WHC and OHC values further suggested that the aerogel possessed a stable but relatively unmodified porous structure. During liquid absorption, the starch matrix may partially expand, resulting in localized distortion of the pore walls and so restricting the overall liquid-holding capacity. Similar behavior has been reported for other starch-based aerogels, where the balance between pore accessibility and structural stability governs fluid retention performance [27].

The mechanical properties of the aerogels were characterized using texture profile analysis (TPA) (Table 2). Among the tested formulations, aerogels prepared from the 2.5 g WCS gel exhibited the highest hardness (approximately 205.67 ± 6.17 N) and springiness (0.85), indicating superior mechanical stability and structural resilience. These results are consistent with the rheological measurements, which demonstrated that the 2.5 g formulation generated the most robust gel network before freeze-drying. A stronger precursor gel is generally capable of better preserving its three-dimensional architecture during ice crystal formation and sublimation, thereby producing aerogels with enhanced mechanical integrity. Liquid absorption significantly influenced the textural behavior of the aerogels. Following water uptake, the porous matrix underwent extensive hydration and swelling, resulting in a pronounced reduction in hardness accompanied by increased springiness. This transition reflects the plasticizing effect of water, which increases molecular mobility within the starch network and promotes hydrogel formation. In contrast, oil absorption mainly occupied the pore spaces without inducing substantial matrix swelling. Consequently, the aerogels retained a relatively rigid structure with limited elastic recovery. These observations indicate that the mechanical response of WCS aerogels is highly dependent on the absorbed medium and is governed by the interactions between the liquid phase and the starch network.

**Table 2:** Textural properties of WCS aerogels after water- and oil-holding treatments.

Sample	Hardness (N)	Springiness (-)
2.5 g WCS Aerogel	205.67 ± 6.17	0.85 ± 0.25
After retaining water	2.45 ± 0.12	0.97 ± 0.02
After applying oil	66.44 ± 14.02	0.39 ± 0.07

Overall, the combination of moderate liquid-holding capacity and favorable mechanical stability suggests that WCS aerogels possess structural characteristics suitable for the incorporation and retention of bioactive compounds. The ability of the aerogels to respond differently to aqueous and lipid environments may also be advantageous for designing controlled-release systems and active food packaging materials.

### 3.5. Structural and Functional Properties of EGC-Loaded Aerogels

#### 3.5.1. Chemical Interactions and Crystalline Structure

**Figure 5:**
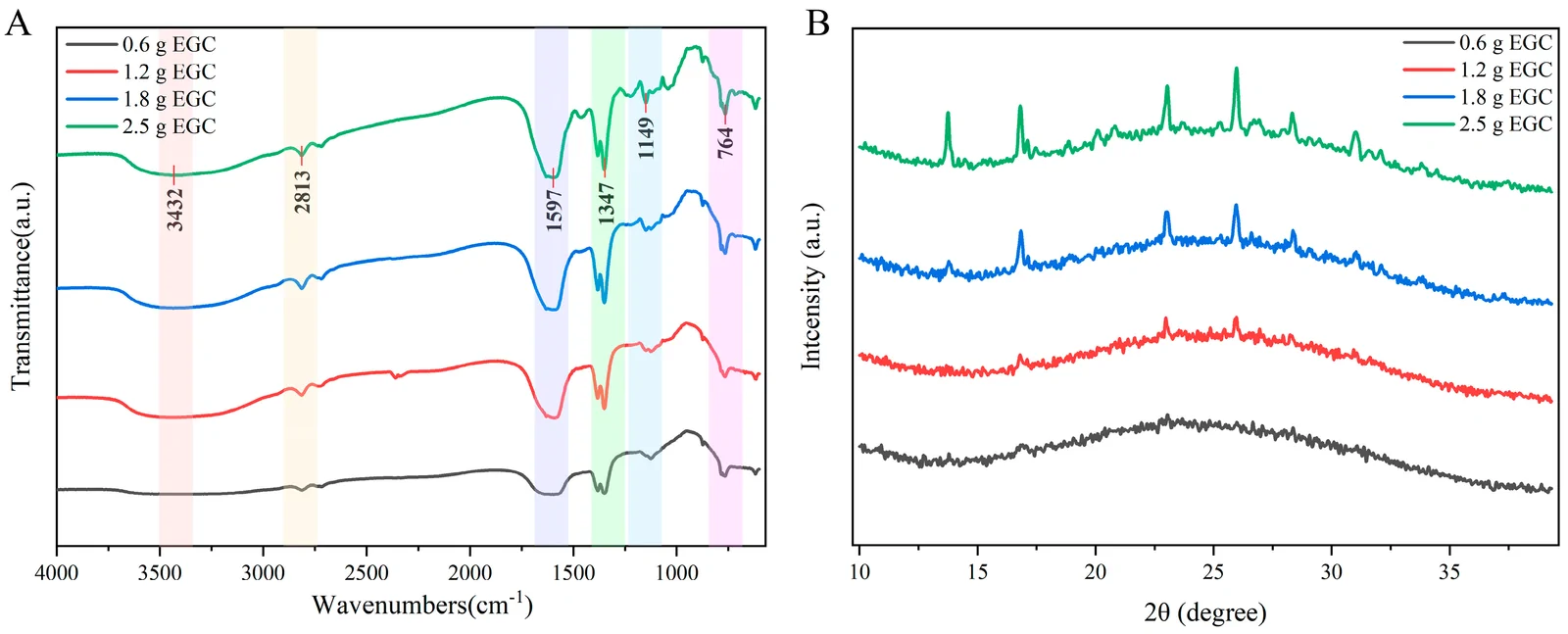
FTIR spectrum (**A**) and XRD patterns (**B**) of EGC-loaded aerogels.

**Figure 6:**
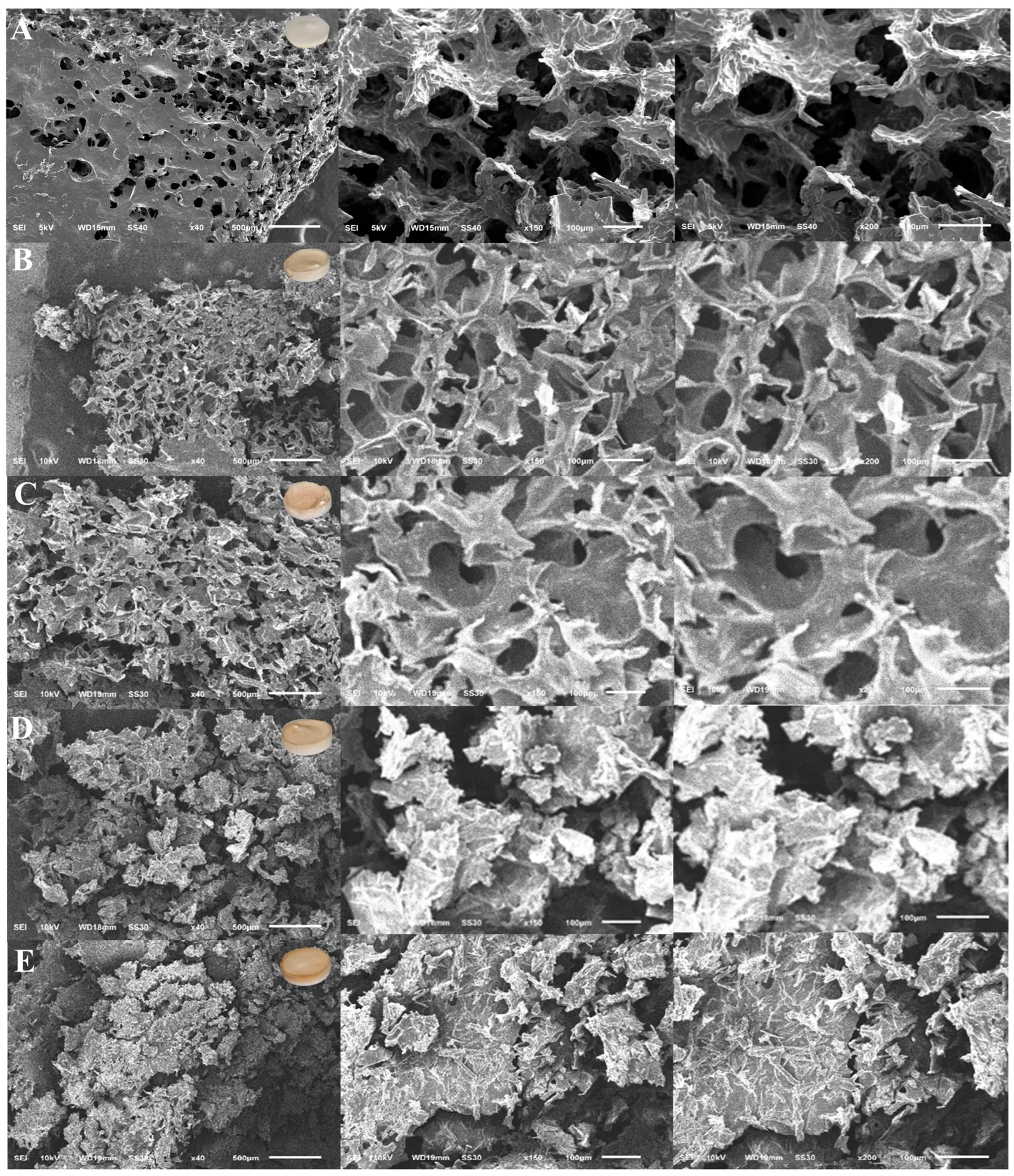
SEM images of EGC-loaded aerogels (images are shown at three magnifications from left to right: 40×, 150×, and 200×). (**A**): Pure WCS aerogel; (**B**): 0.6 g EGC-loaded aerogel; (**C**): 1.2 g EGC-loaded aerogel; (**D**): 1.8 g EGC-loaded aerogel; (**E**): 2.5 g EGC-loaded aerogel).

The molecular interactions between EGC and the WCS matrix were investigated using FTIR and XRD analyses (Figure 5). The FTIR spectrum of pure EGC showed typical polyphenolic absorption bands at 3398, 1630, 1438, and 1140 cm^−1^, corresponding to O–H stretching, aromatic C=C, C–H bending, and C–O–C stretching vibrations, respectively [28]. The FT-IR spectra of WCS granules and WCS aerogels were nearly identical, both exhibiting the characteristic polysaccharide absorption bands at 3442, 2813, 1617, and 1124 cm^−1^ without any impurity peaks (Figure 2), confirming that the aerogel fabrication process did not alter the chemical structure of starch. The spectra of EGC-loaded aerogels exhibited the distinctive bands of both WCS and EGC (Figure 5A), suggesting the effective integration of EGC into the starch matrix. Compared with WCS aerogels (Figure 2), all EGC-loaded aerogels displayed no significant changes in the hydroxyl stretching region (3200–3500 cm^−1^) (Figure 5A). The board O–H absorption band appear to shift from 3442 cm^−1^ (WCS aerogels) to lower wavenumbers (3432 cm^−1^) and exhibited broadening with elevated EGC concentrations (0.6, 1.2, 1.8, and 2.5 g EGC), which may be associated with the development of supplementary hydrogen-bond interactions between the phenolic hydroxyl groups of EGC and the hydroxyl groups of starch chains. The expansion of the O–H stretching band is often considered a possible indicator of hydrogen-bond formation, as the establishment of hydrogen bonds decreases the strength of the O–H covalent bond, thus reducing its stretching frequency. Similar spectral shifts have been reported in starch–polyphenol complexes and are generally considered as potential evidence of intermolecular association [29]. No new characteristic absorption bands were observed after EGC loading (Figure 6A), indicating that the interaction between EGC and WCS was primarily physical rather than involving covalent bond formation. The preservation of the characteristic starch absorption peaks further suggests that the fundamental polysaccharide backbone remained intact throughout the loading process.

XRD analysis revealed that EGC-loaded aerogels exhibited predominantly amorphous diffraction patterns owing to the disruption of native starch crystallinity during gelatinization and freeze-drying (Figure 5B). EGC-loaded aerogels displayed sharp and narrow diffraction peaks in the range of 10–35°, with increased EGC concentration leading to more pronounced peak intensities. Crystals generally display pronounced diffraction peaks, while amorphous materials show broad halos. The pronounced peaks observed in the EGC-loaded aerogels are likely attributed to the crystalline characteristics of EGC [28]. Notably, the maximum intensity was enhanced consistently with higher EGC loading, suggesting that the crystalline regions of EGC were maintained and remained physically dispersed within the amorphous starch matrix without encountering significant structural changes during the aerogel production process. These results may indicate that EGC is present in a crystalline rather than molecularly dispersed state within the aerogel network, and its incorporation does not induce new crystalline ordering of starch chains but rather introduces its own crystalline features into the predominantly amorphous starch matrix.

#### 3.5.2. Morphological Evolution of EGC-Loaded Aerogels

SEM images revealed pronounced changes in the microstructure of WCS aerogels as a function of EGC loading (Figure 6). The pure WCS aerogel had a characteristic freeze-dried porous structure featuring interconnected pore channels and comparatively smooth pore walls (Figure 6A), with a porosity of 51.07% as measured by ImageJ software and a density of 0.0909 ± 0.0051 g/cm^3^.

At 0.6 g EGC loading, the aerogel retained a well-developed three-dimensional porous structure with uniform honeycomb-like pores (Figure 6B). The porosity decreased to 28.66% and density decreased to 0.0645 ± 0.0065 g/cm^3^, suggesting denser pore walls and a lighter aerogel framework. The preserved pore connectivity suggests superior compatibility between EGC and the starch matrix, enabling EGC molecules to distribute uniformly across the network without interfering with aerogel formation. The aerogel loaded with 1.2 g EGC showed a significant degradation of the porous structure (Figure 6C), with porosity further reduced to 18.74% and density of 0.0786 ± 0.0046 g/cm^3^. The interconnected network was replaced by dense and irregular cluster structures, suggesting that the balance between starch–starch and starch–EGC interactions was disrupted at this concentration. Excessive intermolecular forces may have constrained molecular reorganization during the freezing process, thereby inhibiting the formation of a stable ice-templated porous structure. Interestingly, the aerogel loaded with 1.8 g of EGC displayed a more consistent and intricately organized porous structure (Figure 6D). The porosity increased to 36.76% and density increased to 0.0864 ± 0.0029 g/cm^3^, and the pore walls seemed more robust and continuous, suggesting that EGC might function as a secondary cross-linking agent at this concentration. The strengthened hydrogen-bond interactions between EGC and starch chains probably facilitated network stabilization throughout gelation and freeze-drying. Further increasing the loading level to 2.5 g resulted in partial retention of the porous framework with a porosity of 37.69% and density of 0.0898 ± 0.0024 g/cm^3^, along with observable particle accumulations (Figure 6E). These features indicate EGC aggregation and phase separation, suggesting that the loading capacity of the starch matrix had been exceeded. Consequently, excessive EGC disrupted pore formation and reduced structural homogeneity.

Overall, the SEM observations demonstrate that EGC exerts a content-dependent effect on aerogel morphology. The porosity data, calculated using ImageJ software and density data, support these morphological observations: the pure aerogel had the highest porosity (51.07%) and density (0.0909 g/cm^3^), porosity decreased to 28.66% and density to 0.0645 g/cm^3^ at 0.6 g EGC loading, reached a minimum porosity of 18.74% at 1.2 g (with a density of 0.0786 g/cm^3^), then increased to 36.76% (0.0864 g/cm^3^) at 1.8 g and 37.69% (0.0898 g/cm^3^) at 2.5 g. The irregular porosity trend suggests that moderate EGC loading (1.8 g) enhances network structure and porosity recovery, whereas insufficient loading (0.6 g) reduces porosity.

#### 3.5.3. Mechanical and Physical Characteristics

The mechanical properties of the aerogels were strongly influenced by EGC incorporation (Table 3). Compared with WCS aerogel, EGC-loaded aerogels exhibited substantially decreased hardness and springiness. The hardness consistently increased with EGC concentration, whereas springiness decreased. The enhancement in hardness can be attributed to the combination of starch chains and EGC molecules, which increase network density and restrict molecular mobility, resulting in a more rigid and compact structure. However, the increased rigidity reduces the flexibility of the aerogel matrix, leading to decreased springiness. Similar trends have been reported for starch-based systems containing polyphenols [30].

Colorimetric analysis revealed that EGC loading significantly altered the aerogel appearance (Table 3). The color difference (*ΔE*) values ranged from 31.23 to 44.81 compared with WCS aerogel, indicating notable visual changes upon EGC loading. The L value increased from 59.15 at 0.6 g EGC-loaded aerogels to 70.89 at 2.5 g EGC-loaded aerogels, consistent with the white color of pure EGC powder. The a* and b* values changed with EGC loading, with the peak a* (11.72) and b* (20.89) recorded at 1.8 g of EGC-loaded aerogels. However, no consistent concentration-dependent trend was observed for these parameters. The variations in a* and b* values may be associated with the distribution of EGC on the surface or within the interior of the aerogel.

### 3.6. Retention and Protective Efficacy of EGC from Loaded Aerogels

**Figure 7:**
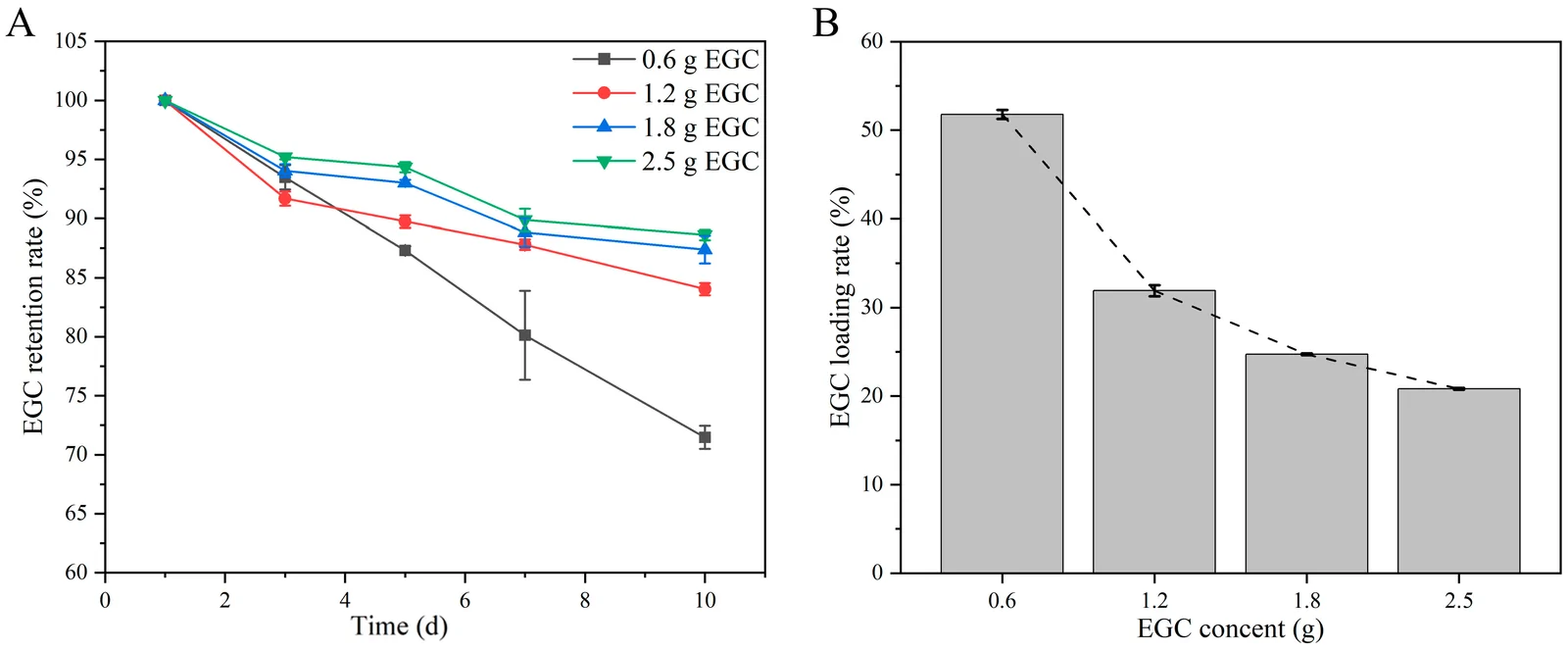
Relationship between EGC retention rate and time (**A**), and EGC loading rate and EGC content (**B**).

The released EGC was quantified against a gallic acid standard curve (0–50 μg/mL, y = 0.01131x − 0.01071, R^2^ = 0.9951) (Figure S3). EGC retention rate and EGC loading rate were evaluated over 10 days (Figure 7). EGC retention rate decreased progressively over time across all loading levels, with higher initial EGC concentrations leading to improved retention (Figure 7A). The 2.5 g EGC-loaded aerogels exhibited the highest retention rate of 88.62 ± 0.45% after 10 days, whereas the 0.6 g EGC-loaded aerogels decreased to 71.47 ± 0.99%, suggesting that higher loading effectively reduced EGC degradation and leakage during storage. The 1.2 g and 1.8 g EGC-loaded aerogels exhibited moderate retention rates of 84.04 ± 0.52% and 87.39 ± 1.16%, respectively, with the 1.8 g EGC-loaded aerogels demonstrating marginally superior stability compared to the 1.2 g EGC-loaded aerogels. The EGC loading rate represents the encapsulation efficiency of EGC within the aerogel matrix on day 1 (Figure 7B). The loading rate diminished gradually with the augmentation of EGC from 51.76 ± 0.51% at 0.6 g EGC-loaded aerogels to 20.82 ± 0.10% at 2.5 g EGC-loaded aerogels. This suggests that although the total content of EGC encapsulated increased with increased loading, the encapsulation efficacy decreased, perhaps due to the restricted binding sites within the starch matrix for polyphenol interactions at higher concentrations, which was consistent with previous research on starch–polyphenol systems, indicating that excessive polyphenol incorporation resulted in molecular clustering and diminished encapsulation efficacy [31].

Overall, the results indicated that WCS aerogels provide substantial protection for EGC, with a 1.8 g loading presenting the ideal optimum between retention stability, encapsulation efficacy, and economization for prolonged storage applications.

### 3.7. Antibacterial Efficacy of EGC-Loaded Aerogels

**Figure 8:**
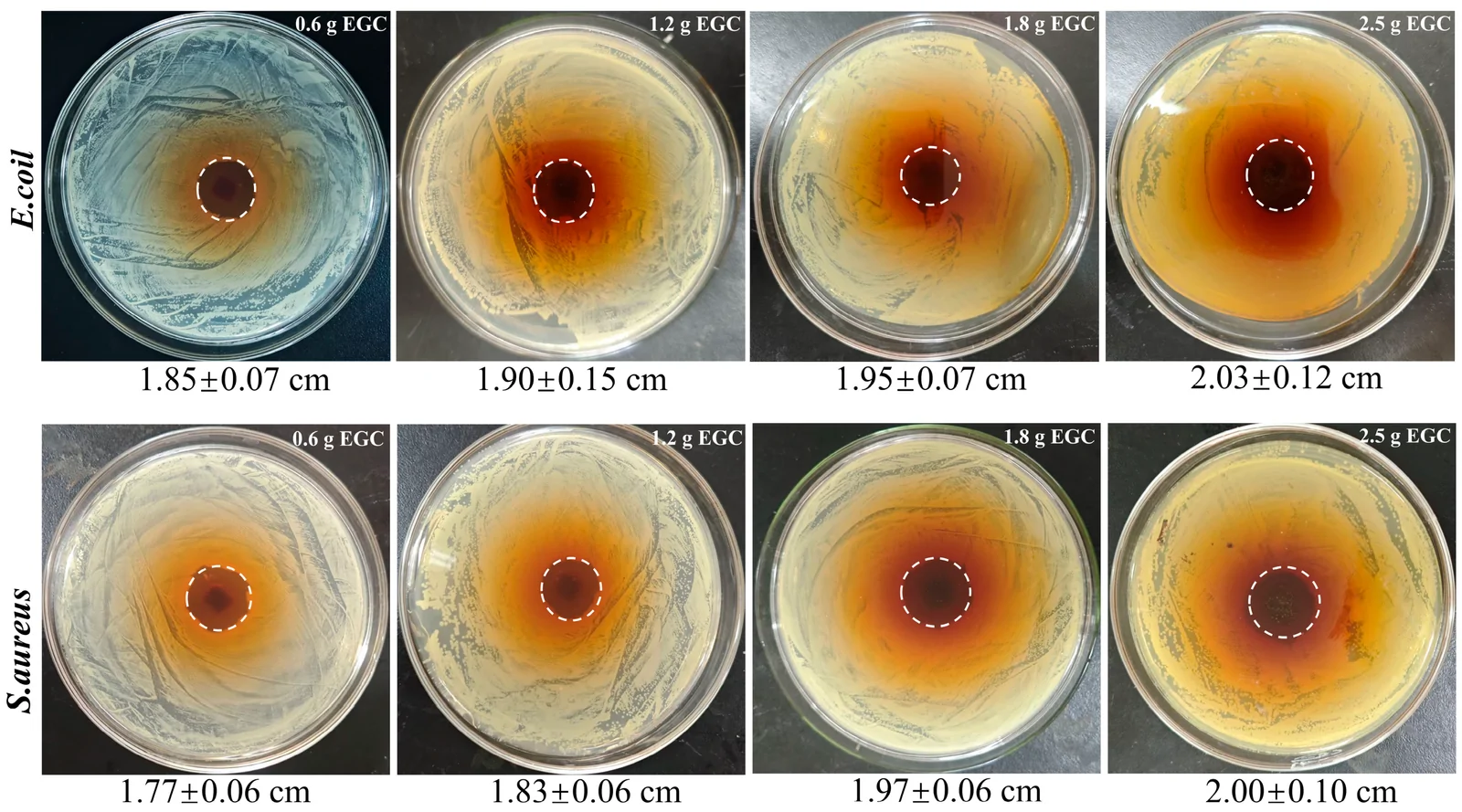
Antibacterial activity of EGC-loaded aerogels.

The antibacterial capacity of EGC-loaded aerogels on *E. coli* and *S. aureus* is shown in Figure 8. All EGC-loaded aerogels produced distinct and transparent antibacterial zones on the plates, confirming that the aerogels possess robust antibacterial capabilities. The 2.5 g EGC exhibited inhibition zone diameters of 2.03 ± 0.12 cm (*E. coli*) and 2.00 ± 0.10 cm (*S. aureus*), while the 0.6 g EGC displayed slightly reduced inhibition zones of 1.85 ± 0.07 cm (*E. coli*) and 1.77 ±0.06 cm (*S. aureus*), respectively. The inhibition zone diameters expanded progressively with EGC concentration, indicating a dose-dependent antibacterial effect. Notably, even the minimal EGC concentration (0.6 g) produced appreciable inhibition zones (1.85 ± 0.07 cm on *E. coli* and 1.77 ± 0.06 cm on *S. aureus*), suggesting that EGC exhibited potent antibacterial activity even at relatively low loading levels. The antibacterial efficacy observed between the minimum and maximum EGC concentrations exhibited a minor disparity, potentially due to the saturation of the antibacterial response at elevated EGC levels, as the release of EGC from the aerogel matrix might reach an effective concentration threshold, beyond which additional increases in loading yield only slight improvements in the inhibition zone diameter.

The increased antibacterial efficacy of the EGC-loaded aerogels is due to the inherent functional groups of EGC, especially the pyrogallol moiety on the B-ring (3′,4′,5′-trihydroxy), combined with the hydroxyls of the resorcinol-type A-ring and the C-3 hydroxyl group [32]. Structure–activity relationship studies have shown that these aromatic hydroxyls, particularly the electron-rich vicinal hydroxyls of the pyrogallol group, are crucial for antibacterial efficacy and remain effective even at low concentrations [32,33,34]. The antibacterial mechanism involves multiple synergistic pathways: (i) hydrogen-bonding and electrostatic interactions with bacterial membranes, compromising their integrity and permeability [32,33,34]; (ii) interference with ribosomal protein synthesis [32]; and (iii) generation of ROS and H_2_O_2_ through autoxidation/redox cycling of the pyrogallol hydroxyls, causing oxidative damage to bacterial cells [33]. Owing to the high potency of these hydroxyl-driven mechanisms, appreciable inhibition zones were observed even at the lowest EGC loading (0.6 g). These synergistic activities combined show broad-spectrum bactericidal activity against both Gram-positive and Gram-negative bacteria [32,33,34], indicating the potential of these EGC-loaded aerogels for natural antimicrobial packaging applications.

### 3.8. Environmental Degradation Characteristics of EGC-Loaded Aerogels

#### 3.8.1. Soil Exposure Examination

**Figure 9:**
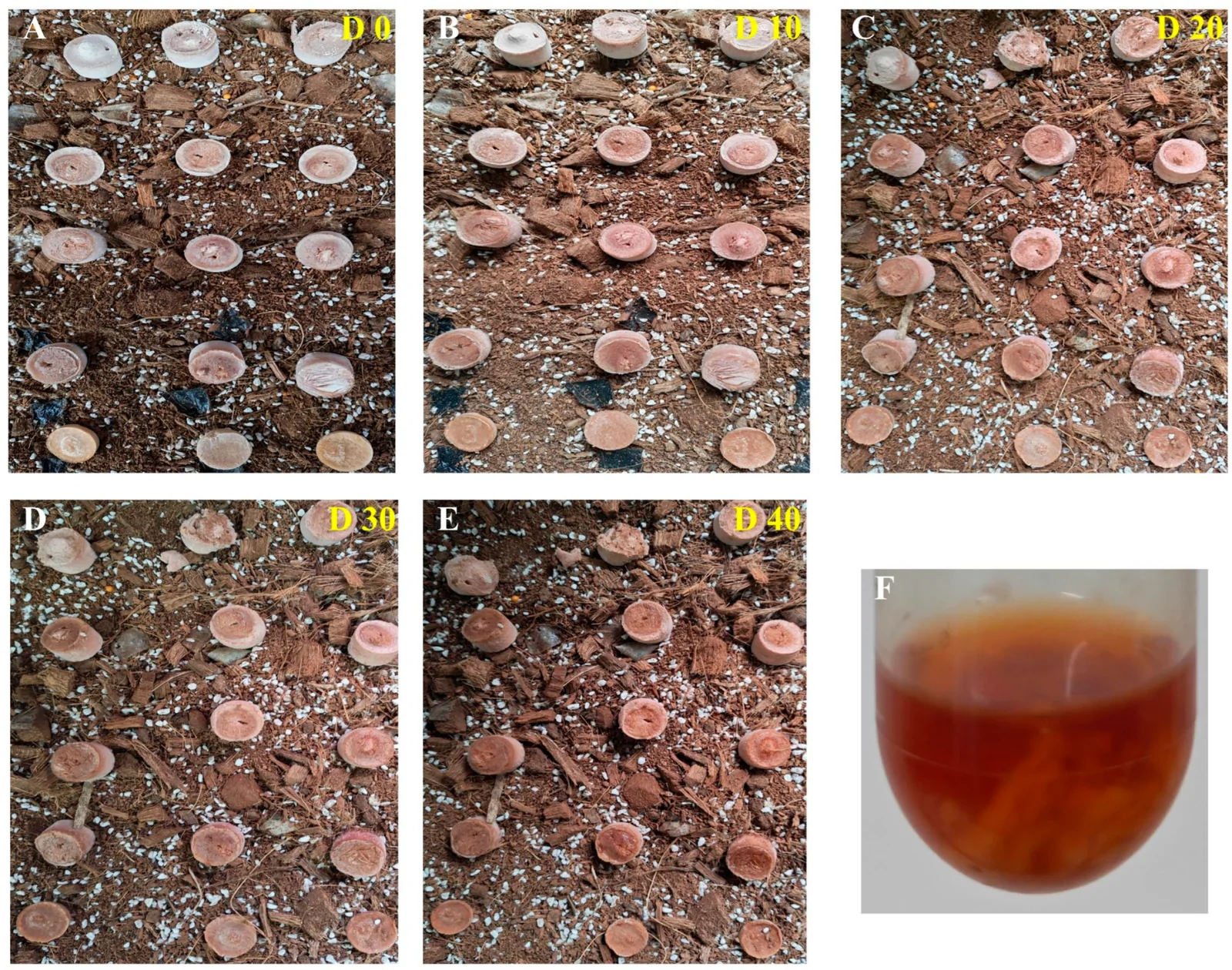
Soil degradation (**A**–**E**) and water solubility (**F**) of EGC-loaded aerogels.

Visual inspection of aerogel samples retrieved at 10-day intervals over a 40-day soil exposure period revealed no evident fragmentation or loss of structural integrity (Figure 9A–E). The only noticeable alteration was a gradual darkening in hue and surface shrinkage, suggesting the adsorption of soil-derived humic compounds and potential surface oxidation of the absorbed EGC, which showed early-stage aging and structural deterioration. The minimal mass reduction and insignificant structural degradation observed over this period may be attributed to two possible factors. First, while the starch-based matrix is inherently biodegradable, the addition of EGC may influence the activity of starch-degrading soil microorganisms, potentially modulating the early-stage degradation process. Second, a 40-day exposure duration is comparatively short for considerable degradation of a macroscopic porous biopolymer monolith, as starch-based materials typically require prolonged exposure to natural environmental conditions for meaningful fragmentation and mineralization to be observed. Overall, these results indicate that although the aerogel system is constructed from a biodegradable polymer backbone, its degradation behavior under soil conditions requires further systematic evaluation.

#### 3.8.2. Water Solubility and Dispersion Assessment

In contrast to the comparatively sluggish disintegration noted under soil exposure circumstances, the aerogels demonstrated a fast reaction when exposed to an aquatic environment. After immersion in deionized water, the EGC-loaded aerogels exhibited rapid swelling, subsequent structural dispersion, and eventual breakdown (Figure 9F), driven by the hydrophilic properties of the starch backbone and the highly porous structure, creating favorable conditions for subsequent microbial colonization, which indicated that the aerogel substance exhibits remarkable water solubility.

#### 3.8.3. Integrated Degradation Profile and Implications

The amalgamation of findings from both environmental assays yields a thorough comprehension of the end-of-life behavior of the WCS/EGC aerogels. The aerogel exhibited two separate reactions: rapid physical dissolution in water and slow surface deterioration in soil exposure. The examination of soil exposure revealed that the aerogel surface shrank and darkened without compromising its structure, indicating early-stage aging. The rapid dispersal of aerogels in water is a disintegration driven by expansion and chain relaxation.

The role of EGC in aerogels was substantial. In addition to providing functional characteristics like antioxidant and antibacterial effects upon application, EGC may also postpone the degradation of microorganisms. This dual functionality established a realistic temporal equilibrium: the material maintained sufficient structural and functional integrity throughout its intended service life (e.g., as a fresh-food packaging pad), while also maintaining the inherent capacity for complete post-use breakdown. These attributes collectively highlight its potential as a sustainable alternative to traditional plastics, addressing both functional performance standards and end-of-life environmental concerns.

## 4. Discussion

This comprehensive analysis demonstrates that the incorporation of EGC into WCS aerogels is not merely a physical filling process but a complex modulation of the material’s multi-tiered architecture, which subsequently governs its macroscopic properties. The non-monotonic fluctuations observed in structural, mechanical, and functional metrics with respect to EGC concentration can be logically explained by changes in molecular contacts and their influence on network formation (as illustrated in Figure 5).

### 4.1. EGC-Mediated Structural Evolution: From Disruption to Reinforcement

The primary observation is the concentration-dependent, non-monotonic development of the aerogel’s porous microstructure (Section 3.5.2, Figure 6). At a low concentration (0.6 g EGC), EGC molecules are expected to be evenly distributed and largely engage with starch chains by hydrogen bonding, without substantially disrupting the pre-existing starch–starch network established during gelatinization. This produces a durable, highly interconnected porous framework.

The porosity data revealed that the pure WCS aerogel exhibited the highest porosity (51.07%), which decreased to 28.66% at 0.6 g EGC loading, reached a minimum of 18.74% at 1.2 g, and then recovered to 36.76% at 1.8 g and 37.69% at 2.5 g. The sharp decline in porosity at 1.2 g EGC loading is noteworthy. We hypothesize that at this intermediate concentration, EGC molecules function as competitive disruptors. The numerous phenolic hydroxyl groups of EGC may selectively engage in hydrogen bonding with starch chains, thereby disrupting the intermolecular and intramolecular hydrogen bonds crucial for forming a continuous, resilient three-dimensional starch gel network during the sol–gel transition. The degraded gel network, when subjected to freeze-drying, results in the observed thick, aggregated shape with diminished porosity. This aligns with the rheological premise that a favorable equilibrium of polymer–polymer and polymer–solute interactions is essential for robust gelation.

In contrast, at a loading of 1.8 g EGC, a more structured and resilient porous network is obtained with porosity recovering to 36.76%. This signifies a shift in the function of EGC from a disruptor to a multifunctional cross-linker. At elevated EGC concentration, the system may attain a percolation threshold where EGC-EGC interactions, such as π-π stacking of aromatic rings, become prominent in conjunction with EGC-WCS hydrogen bonds. This synergistic interaction may enable the creation of a secondary, reinforcing network in which EGC clusters serve as supplementary cross-linking nodes. This novel structure not only reinstates structural integrity but also improves the uniformity and mechanical stability of the pore walls, as seen by the enhanced hardness and springiness (Section 3.5.3).

The structural degradation at 2.5 g EGC is ascribed to phase separation and excessive aggregation. The solubility threshold of EGC inside the starch matrix is surpassed, resulting in the development of substantial EGC aggregates that obstruct pore formation and generate discontinuous, granular domains, ultimately compromising the integrity of the coherent aerogel network.

### 4.2. Integration of Mechanical Response and Release

The mechanical and release characteristics are direct results of the previously indicated structural evolution. The function of EGC as a rigid cross-linker is clearly revealed by the texture profile analysis (Section 3.5.2). The consistent rise in hardness and decline in springiness/cohesiveness with EGC loading correspond to the augmented density of hydrogen-bonded cross-links, which impede chain mobility and bolster network rigidity, compromising its elastic recovery capacity.

This reinforced and densified network at optimal loading (1.8 g EGC) is precisely the key to the controlled release behavior (Section 3.6). The identified critical release window (0.6–1.2 g) marks the transition from a release-dominant regime to a retention-dominant regime. Below this window, the network is relatively open, allowing for faster diffusion and higher cumulative release. As the loading increases into the optimal range (~1.8 g), it is possible that two factors synergistically retard release: (i) the increased diffusion path tortuosity and decreased effective pore size of the reinforced network, and (ii) the stronger binding affinity of EGC molecules entrapped within the dense hydrogen-bond network. The saturation of release rate reduction beyond 1.2 g suggests that the diffusion-limiting effect of the matrix approaches its maximum, aligning with the observation of optimal network formation at 1.8 g loading.

### 4.3. Functional Performance and Environmental Fate: A Balanced Design

The operational effectiveness and ecological characteristics of the composite aerogels arise from this meticulously calibrated structure. The antibacterial activity (Section 3.7) demonstrated a distinct dose–response relationship, facilitated by the aerogel’s capacity to sustainably release bioactive EGC from its porous reservoir, rather than in a sudden burst. The optimal 1.8 g EGC-loaded aerogels offer a favorable balance of adequate active agent loading and a suitable release rate to sustain an effective antibacterial concentration throughout time.

The degradation tests (Section 3.8) revealed two distinct physical responses of the EGC-loaded aerogels under different environmental conditions: rapid disintegration in aqueous environments and gradual surface aging under soil exposure. In the soil exposure test, the aerogels exhibited surface shrinkage and darkening over 40 days without significant structural collapse, indicating early-stage aging rather than complete biodegradation. The water-induced dispersion, driven by swelling and chain relaxation, reflects the physical instability of the hydrophilic starch matrix under wet conditions, which may facilitate subsequent microbial reproduction. This combination attains an effective equilibrium between stability during use and degradability after use.

## 5. Conclusions

The characteristics of EGC-loaded WCS aerogels are determined by a concentration-dependent interplay of molecular interactions. The 1.8 g EGC loading emerged as the optimal formulation, producing an aerogel with improved porosity (36.76%), the highest hardness (55.27 N), and a sustained release profile with approximately 80.5% retention after 10 days. EGC-loaded aerogels exhibit antibacterial activity against *E. coli* and *S. aureus*. EGC-loaded aerogels provide decreased mechanical strength, a diffusion-managed release profile for extended antibacterial effectiveness, and a regulated degradation timeline. This study establishes a comprehensive framework for designing starch-based active packaging materials, integrating the bioactive ingredient as a core structural component to enable tailored multifunctional capabilities.

## Figures and Tables

**Table 1:** Hydrolysis properties of WCS aerogels.

WCS Powder/g	Hardness (N)	Springiness (-)	Water Absorption Rate (%)	Oil Absorption (%)
2	48.00 ± 2.82	0.55 ± 0.13	5.62 ± 1.37	4.16 ± 1.33
2.5	205.67 ± 6.17	0.85 ± 0.25	5.29 ± 1.65	4.51 ± 1.42
3	179.52 ± 11.99	0.45 ± 0.03	5.25 ± 1.15	4.40 ± 0.92

Hardness is defined as the peak force required to achieve a predetermined deformation during the first compression cycle, reflecting the resistance of the aerogel matrix to compressive stress. Springiness, defined as the ratio of the sample’s height recovery during the second compression cycle to its original height during the first compression cycle, measures the elastic recovery capacity of aerogel.

**Table 3:** Characterization of EGC-loaded aerogels.

EGC-Loaded Concentration/g	Hardness (N)	Springiness (-)	L* (Lightness)	a* (Red-Green Value)	b* (Yellow-Blue Value)	*ΔE*
0	205.67 ± 6.17	0.85 ± 0.25	90.21 ± 0.86	1.13 ± 0.19	6.12 ± 0.72	0
0.6	24.12 ± 0.27	0.22 ± 0.03	59.15 ± 2.08	11.65 ± 0.21	18.33 ± 0.97	44.81 ± 1.87
1.2	31.58 ± 1.87	0.18 ± 0.02	66.54 ± 1.13	9.09 ± 0.66	19.16 ± 0.99	38.13 ± 1.08
1.8	47.70 ± 0.40	0.10 ± 0.01	66.90 ± 2.52	11.72 ± 0.39	20.89 ± 1.42	39.43 ± 2.15
2.5	55.27 ± 0.90	0.08 ± 0.02	70.89 ± 4.80	6.69 ± 1.12	13.37 ± 2.29	31.23 ± 4.36

*Δ*E values for EGC-loaded aerogels are calculated relative to the pure WCS aerogel as the control (set to 0).

## Data Availability

The original contributions presented in this study are included in the article/Supplementary Materials. Further inquiries can be directed to the corresponding authors.

## Supplementary Materials

The following supporting information can be downloaded at https://www.mdpi.com/article/10.3390/foods15183320/s1, Figure S1. Extraction of WCS; Figure S2. Amylose standard curve; Figure S3. Gallic acid standard curve.
