# Supplementary material for: Development of Epigallocatechin-Loaded Water Caltrop Starch Aerogels: Structure, Functional Properties and In Vitro Release Behavior
Source: Foods. 2026 Sep 19;15(18):3320. doi: 10.3390/foods15183320 (PMC13606700; doi:10.3390/foods15183320)
Supplement: Supplementary file 1 [file foods-15-03320-s001.zip › foods-4450000-supplementary.pdf]

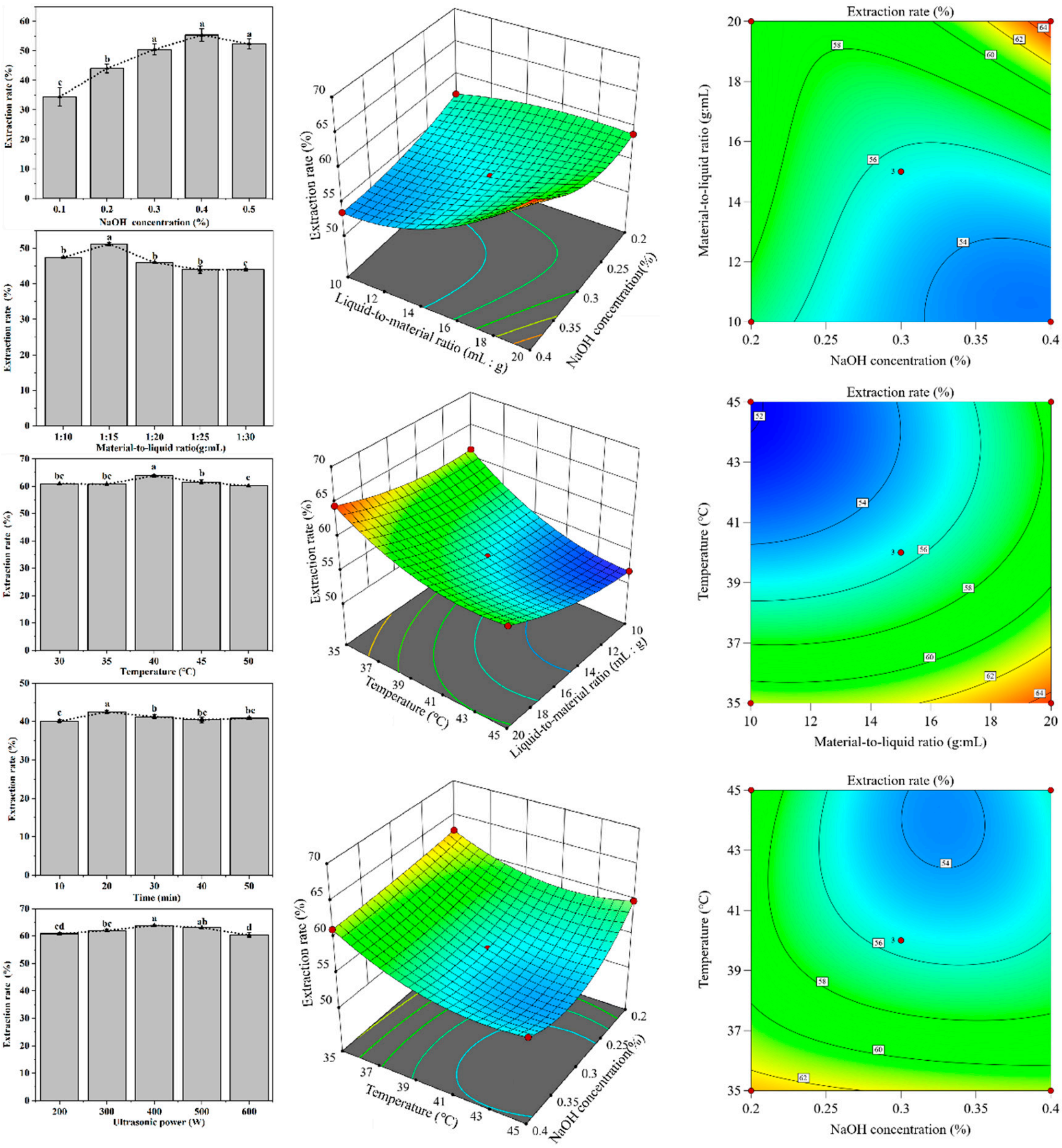

Figure S1. Extraction of WCS

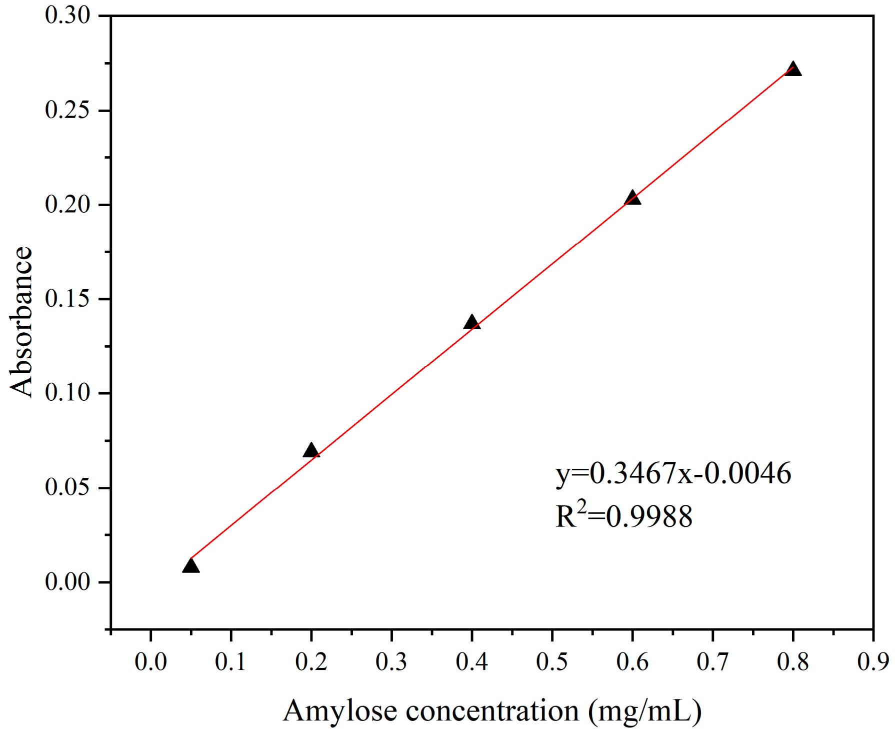

Figure S2. Amylose standard curve

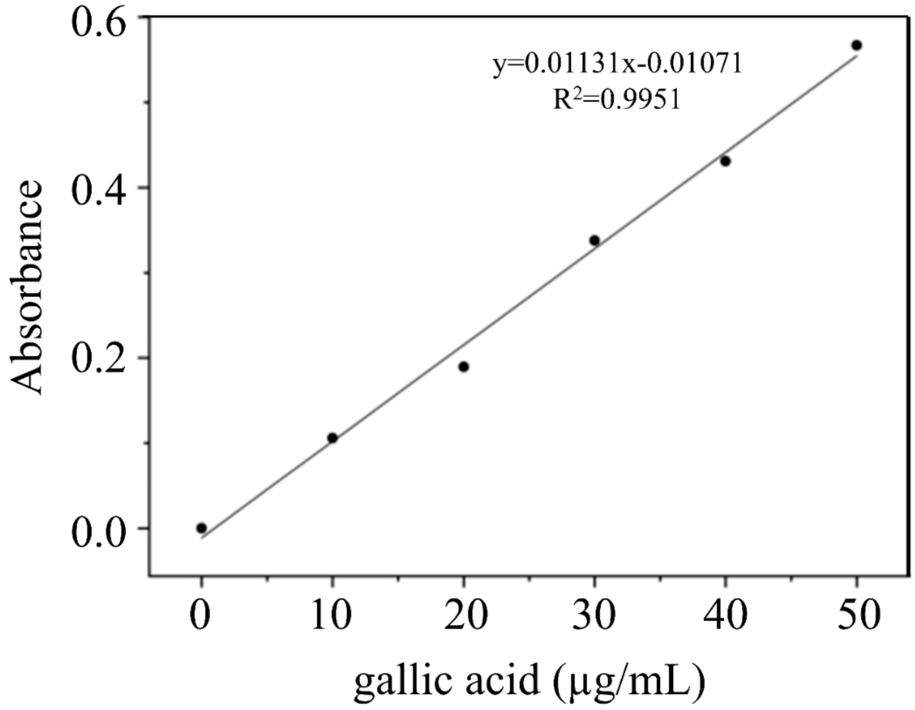

Figure S3. Gallic acid standard curve
